# Evidence on the contribution of community gardens to promote physical and mental health and well-being of non-institutionalized individuals: A systematic review

**DOI:** 10.1371/journal.pone.0255621

**Published:** 2021-08-06

**Authors:** Tarsila Lampert, Joana Costa, Osvaldo Santos, Joana Sousa, Teresa Ribeiro, Elisabete Freire

**Affiliations:** 1 Instituto de Saúde Ambiental, Faculdade de Medicina, Universidade de Lisboa, Lisboa, Portugal; 2 EnviHeB Lab, Instituto de Saúde Ambiental, Faculdade de Medicina, Universidade de Lisboa, Lisboa, Portugal; 3 Unbreakable Idea Research, Lisboa, Portugal; 4 Laboratório de Nutrição, Faculdade de Medicina, Universidade de Lisboa, Lisboa, Portugal; 5 Câmara Municipal de Cascais, Lisboa, Portugal; 6 Departamento das Ciências Sociais do Território, Faculdade de Arquitectura, Universidade de Lisboa, Lisboa, Portugal; University of Hradec Kralove: Univerzita Hradec Kralove, CZECH REPUBLIC

## Abstract

**Introduction:**

There has been growing interest in community gardens as an effective and affordable health promotion strategy. However, most available evidence is derived from qualitative studies, whereas quantitative research on this subject is limited.

**Objectives:**

To synthetize the literature about physical and mental health outcomes associated with community gardening. Two main questions were addressed: a) is there evidence, from quantitative studies, that community gardening is associated to physical and mental health and well-being of non-institutionalized individuals? b) Does community gardening provokes any discomfort in terms of physical health, i.e., bodily pain, to their beneficiaries?

**Methods:**

A systematic review of the literature was carried out following PRISMA guidelines by searching relevant electronic databases (PubMed, Scopus, and Web of Science). Empirical, quantitative studies published in English with no restrictions concerning the date of publication were considered eligible. The quality of the evidence was appraised using the tool developed by the National Heart, Lung, and Blood Institute of the National Institutes of Health for Observational Cohort and Cross-Sectional Studies.

**Results:**

Overall, 8 studies were considered eligible, of which seven studies were rated as having good methodological quality (one scored as fair). Community gardeners had significantly better health outcomes than their neighbours not engaged in gardening activities in terms of life satisfaction, happiness, general health, mental health, and social cohesion.

**Conclusion:**

Community gardens are associated to health gains for their users, irrespective of age, being an affordable and efficient way of promoting physical and mental health and well-being. To encourage the design, maintenance, and prospective evaluation of supportive urban environments promoting healthy and, at the same time, sustainable lifestyles, is essential to achieve public health gains and environmental sustainability.

## Introduction

The global burden of mental illness is considerable, and it encompasses individual, family, social and economic impacts [1]. At the individual level, people suffering from (transient or chronic) mental illness also experience impaired quality of life characterized by distress-related feelings, lack of control, low self-esteem and confidence, among others [2, 3]. This condition strongly affects their everyday living [4], including their social interactions [5] and performance at the workplace [6]. Moreover, stigma and discrimination towards people with mental illness still prevails. with negative consequences for those mentally ill [7], who might refrain from seeking professional help [8].

A recently published literature review concluded that the global burden of mental illness in terms of years lived with disabilities (YLDs) has been underestimated, and placed mental illness at the top of the list accounting for 32.4% of YLDs [1]. Concerning disability-adjusted life-years (DALYs), mental illness is at the same level as cardiovascular and circulatory diseases, accounting for 13.0% of DALYs [1]. These pictures call for action against the high burden of mental illness and gain urgency in the context of the current COVID-19 pandemic. The available literature addressing the impact of COVID-19 on mental health supports psychological suffering (e.g., anxiety, depression, post-traumatic disorder, psychological distress) from lockdowns, social distancing measures, being diagnosed with COVID-19 or being a health professional working at the frontline [9–11]. Now more than ever before, mental health promotion should be the main avenue to tackle the burden of mental illness.

Human contact with nature has been highly valued in health promotion over the last years. As such, there has been a growing interest on the health benefits from greenspace exposure, i.e., parks, gardens and forests, with evidence in favour of positive health outcomes (e.g. [12–17]). Interestingly, some authors argue that the mental health benefits arising from the contact with nature should embody the list of services provided by the natural ecosystems [13], which include crop pollination and climate regulation, among others. Empirical evidence supports the beneficial influence of greenspace exposure on several health outcomes. These include physical and general health [18]; disease prevention [19–21]; restoration of the individuals’ psychological resources by providing them with an environment free from physical and social stressors [22]; and improvement of the cognitive function, including memory, attention, concentration and impulse inhibition [23].

Contact with nature in urban areas is challenging, because outdoor greenspaces are much reduced compared to non-urban, rural areas. Cox et al. (2017) investigated which natural characteristics of selected neighbourhoods in British urban areas contributed the most for mental health gains of the nearby residents. These authors concluded that vegetation cover and the abundance of birds in the afternoon were the most relevant factors contributing for mental health benefits measured as decreased prevalence of depression, anxiety, and stress. Another study concluded that the prevalence of mental health conditions can be reduced if minimum values of vegetation cover are maintained [20]. Thus, green spaces can also function as a promotion strategy for mental health [24]. These findings are highly relevant to inform strategic public health interventions and support urban planning solutions that ease the interaction between city dwellers and nature [25].

In 2019, approximately 57% of the world population lived in cities [26] and spent the great majority of the time indoors (e.g., at home, school, workplace); pre-COVID-19 pandemic estimates pointed out that humans spend, on average, 85–90% of their time indoors [27]. Then, the great challenge is to integrate nature within the urban infrastructure. One avenue to tackle this issue is by promoting citizens’ participation in community gardens [28]. Community gardens are also known as urban gardens, allotment gardens, allotments, community agriculture, agricultural allotments, roof top gardens, roof top agriculture, roof top farms, all these terms referring to a greenspace located in an urban area, where community residents mainly grow vegetables for their own consumption, although border flower beds are also commonly grown, while profiting from it in the company of other members from the neighbourhood and/or their family with no imposed frequency schedule [29]. Community gardens serve various relevant functions at multiple levels. At the environmental level, they can add to climate change mitigation by sequestrating atmospheric carbon, thus contributing for reducing the amount of greenhouse gases [30]. As previously mentioned, community gardens are also considered a sustainable way to improve the quality of life of city dwellers [31, 32], namely by providing citizens with the opportunity to be in close contact with nature [33, 34] while supporting healthy lifestyles [35].

Horticultural therapy, i.e., the engagement of individuals in horticultural activities with live plants to improve their health and well-being [36], has produced health benefits on people with various mental health conditions in different settings (e.g., [37–39]. However, less is known about the mental health outcomes for non-clinical populations engaging in gardening activities. A study carried out in The Netherlands provided support for a positive effect of gardening activities on relief from acute stress [40]. In another study, community gardeners were induced some stress and randomly assigned to a 30-min outdoors gardening session or indoors reading. The levels of stress measured as salivary cortisol and self-reported positive mood were significantly lower in those assigned to gardening activities *versus* the reading group [40]. There is also some evidence that engaging in community gardening improves well-being by encouraging healthy behaviours, such as physical activity [41] and the consumption of locally grown healthy foods [42, 43]. Moreover, a qualitative study conducted in the United States pointed out that gardening is considered a moderate intensity activity that can provide older adults with the health benefits of regular moderate intensity physical activity [44]. On the other hand, some body positions during gardening can be uncomfortable or even cause pain when the target audience is the elderly [44].

Despite increased attention that community gardening has received in recent years, most available evidence on health and well-being promotion comes from qualitative studies [45, 46]. As such, this study aims to review quantitative evidence about physical and mental health outcomes of community gardening. More specifically, this literature review addresses two main questions. First, is there evidence, from quantitative studies, that community gardening contributes to increased physical and mental health and well-being of non-institutionalized individuals? Second, does community gardening provokes any discomfort in terms of physical health, i.e., bodily pain, to their users? To answer these questions, a systematic literature review following PRISMA guidelines [47] was conducted.

## Methods

### Search strategy and inclusion criteria

A systematic literature review was performed following PRISMA guidelines [47] through a search of studies contained in PubMed, Scopus and Web of Science electronic databases with no restrictions concerning publication date (PRISMA Checklist is provided as S1 Checklist). The search was conducted on July 2–4, 2019, and updated on November 17–19, 2020, by using a pairwise combination of two blocks of both free-text and medical subject headings (MeSH) terms. The search strategy followed for PubMed is provided as S1 File. The following keywords were used as alternatives: (“Community garden*” OR “Urban garden*” OR “Allotment garden*” OR Allotment OR “Community agriculture” OR “Agricultural allotment” OR “Roof*top garden*” OR “Roof*top agriculture” OR “Roof*top farm*”) AND (“Mental health” OR “Quality of life” OR *happiness OR “Well*being” OR “Life satisfaction” OR “Satisfaction with life” OR “Psychological well*being” OR “Subjective well*being” OR Depression OR Anxiety OR Dysthymia OR Loneliness OR “Musculoskeletal injur*” OR “Musculoskeletal condition*” OR “Osteo*articular injur*” OR “Osteo*articular disease*”).

Citations retrieved were downloaded, duplicates were removed, titles and abstracts were independently screened for eligibility by two authors of this review (TL and JC). In case of disagreement, a third researcher (OS) independently assessed the article for eligibility. Articles were assessed for eligibility based on the following criteria: a) empirical cross-sectional quantitative studies; b) community-based studies; c) data on subjective or psychological well-being and/or physical well-being reported in the study; d) the gardens referred to in the studies were exclusively community gardens; and e) full texts available in English. Documents reporting data from studies conducted in home gardens, also referred to as household gardens, as well as qualitative studies, literature reviews and grey literature were excluded.

### Data extraction and analysis

Data were independently extracted by two authors of this review (TL and JC) into a standardized table, and a third researcher (OS) checked data for consensus. Data extracted from each article were as follows: authors, year of publication, title of the paper, country of data collection, setting (rural versus urban), target population, sample size of the participants, sample size of gardens, inclusion criteria, exclusion criteria, characteristics of the gardens (e.g., area, number of plots), motivation(s) for selecting those gardens, health outcomes under study (i.e., subjective or psychological well-being and/or physical well-being), instruments of data collection, main conclusions, and direction of the association between community gardening and health outcomes.

### Quality assessment

The quality of the evidence was appraised using the tool by the National Heart, Lung, and Blood Institute of the National Institutes of Health (NIH) for Observational Cohort and Cross-Sectional Studies [48]. This was done independently by two authors of the paper (TL and JC); in case of disagreement, an independent evaluation was made by a third researcher (OS).

## Results

**Fig 1:**
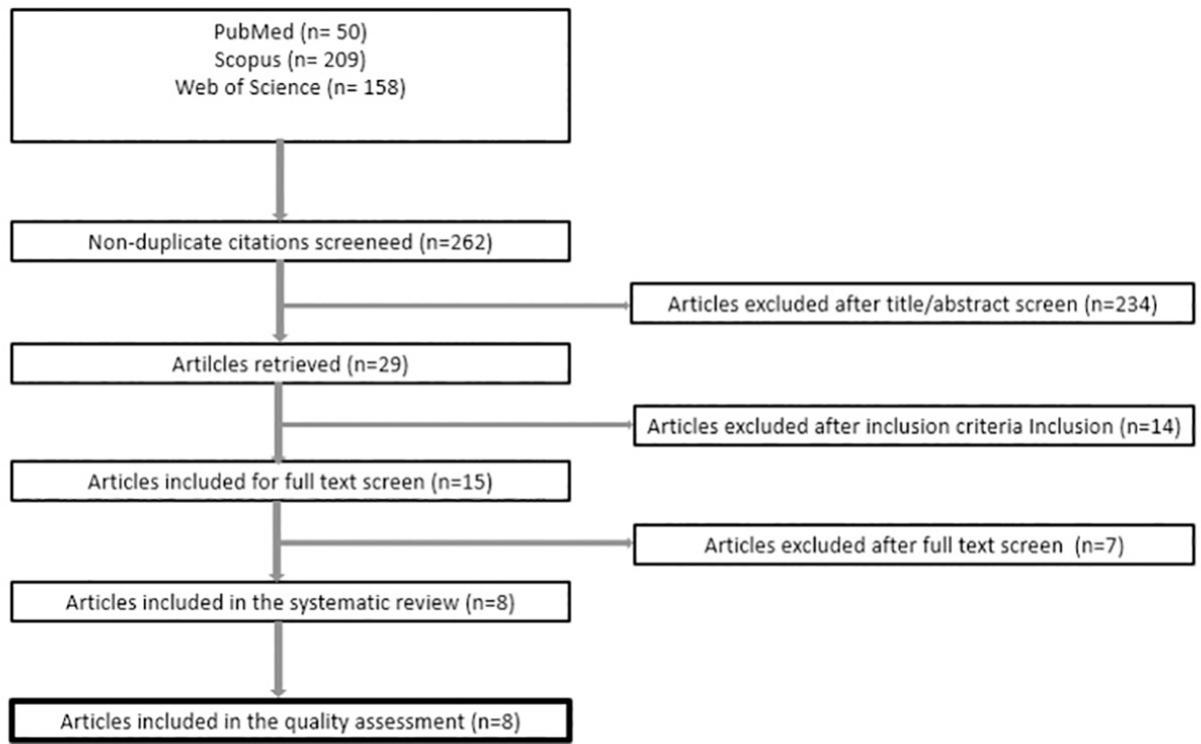
Preferred Reporting Items for Systematic Reviews and Meta-Analyses (PRISMA) fluxogram of study selection.

Fig 1 depicts the selection process of articles included in this systematic literature review. Eight articles were considered eligible from the initial list of 262 potentially relevant titles. Main methodological characteristics of the articles are summarized in Table 1. Studies included in this literature review were conducted in the United States of America (n = 3), United Kingdom (n = 1), the Netherlands (n = 1), Japan (n = 1), Singapore (n = 1), and Portugal (n = 1). All studies were conducted in an urban setting and had a cross-sectional design; one of them used a mixed-methods approach by combining cross-sectional quantitative data collection and qualitative semi-structured interviews [49]. Target population was composed of adult gardeners and non-gardeners residing in the cities where the studies were carried out; one study targeted Bhutanese refugees living in the United States [49]. In all studies, outcomes of interest were compared between gardeners and non-gardeners. With regard to inclusion and exclusion criteria, these were generally not provided in the articles, with three exceptions in which specific inclusion criteria for the target population were defined: a) individuals aged 50+ years [50], b) Nepali Bhutanese Refugees [49], and c) gardeners from the urban organic allotment garden at Devesa Park, Portugal [51]. Only the study carried out in Singapore referred to exclusion criteria: participants who did not complete the survey; individuals under the age of 18 and over the age of 100; and residents who engaged in physical activities outdoors, alone and not in a group, were excluded from the study [52]. The number of community gardens analyzed in the studies ranged from 1 to 64; however, not all studies reported this information. Community gardens were variable in terms of their characteristics, including size and facilities offered to gardeners. Detailed information regarding garden characteristics was generally not provided in the papers (Table 1).

**Table 1:** Characteristics of the community gardens described in the studies included in this systematic literature review.

Reference	Country of data collection	Setting	Target population	Participants’ inclusion criteria	Community Gardens	
					Sample size	Characteristics
Blair et al., 1991 [54]	United States of America	Urban	Gardeners from the Philadelphia Urban Gardening Project and non-gardeners from the same geographical area.	NS	64	NS
van den Berg et al., 2010 [40]	The Netherlands	Urban	Members of 12 allotment sites and their neighbours.	NS	12	Average area of 7 ha.
Hawkins et al., 2011 [50]	United Kingdom	Urban	Individuals aged 50 and over, members of various indoor and outdoor activity groups.	Aged 50 years and over	NS	NS
Gerber et al., 2017 [49]	United States of America	Urban	Bhutanese refugees.	To be a Bhutanese refugee living in the USA	2	The majority of plots required some form of transportation to reach.
Soga et al., 2017b [19]	Japan	Urban	Nerima city residents.	NS	24	Area of allotment sites ranged between 0.05 and 0.47 ha.
Booth et al., 2018 [53]	United States of America	Urban	Residents in a disadvantaged neighbourhood.	NS	4	Total area of the community gardens of about 3.2 ha.
Mourão et al., 2019 [51]	Portugal	Urban	Gardeners from Devesa Park.	To be a gardener from the urban organic allotment garden at Devesa Park	1	The allotment gardens included 192 family plots of 25m^2^ /plot, 6 raised plots of 4m^2^ /plot, 3 plots of 100 m2/plot and a common composting area (120 m^2^), 6 tool houses, 40 water taps, rest and snack areas and sanitary equipment.
						The urban organic allotment gardens are integrated in the park green area; access only granted for gardeners.
Koay & Dillon, 2020 [52]	Singapore	Urban	Community dwellers residing and engaging in gardening or outdoor activities in Singapore	NS	NS	NS

Notes: NS = Not specified.

### Characterization of the participants

Characterization of the participants (gardeners and non-gardeners) and data on the association between gardening and mental and physical well-being are provided in Table 2. The sample size of community gardeners ranged from 16 [53] to 165 [19], whereas the number of participants enrolled in the studies who were not engaged in gardening activities ranged from 28 [49, 52] to 167 [19]. One study considered two groups of participants, i.e., regular and occasional gardeners, based on the frequency they engaged in gardening activities [53]. Two studies also included a group of people who performed their gardening activities within their home gardens [50, 52]. With regard to non-gardeners, one study addressed community gardening and other leisure activities for stress reduction, and the latter group included home gardeners, walkers and people who engaged in physical activity indoors [50].

**Table 2:** Characteristics of the participants, i.e., gardeners (G) and non-gardeners (NG), outcomes and conclusions of the studies included in this systematic review of the literature.

Reference	Participants			Outcomes measured		Instruments used for data collection	Main conclusions	Direction of the association
	Sample size	Sex (%, female)	Age (mean ± SD)	Physical health & well-being	Psychological wellbeing, Subjective Wellbeing and Psychossocial indicators			
Blair et al., 1991 [54]	G: 144 NG: 67	G: 53.8% NG: 65.7%	G: 60.3 ± 15.1 NG: 45.5 ± 15.3	• Self-reported health	• Life satisfaction	NS	• Gardeners reported significantly higher life satisfaction and positive life events than non-gardeners. • Participants in gardening activities were more actively involved in community projects than their neighbours.	Positive
Van den Berg et al., 2010 [40]	G:121 NG:63	G:47.1% NG: 58.7%	G: 61.5 ± 11.8 NG: 55.9 ± 13.8	• Perceived general health • Acute health complaints • Physical constraints • Chronic illnesses • Healthcare use	• Perceived stress, life satisfaction, loneliness, and social contacts with friends	• Short Form Health Surveys-36 (SF-36) • Life Satisfaction Index-8 • Self-reported levels of physical activity (SQUASH)	Impacts of community gardening on health and well-being were moderated by age: older gardeners (+62 years) scored better for all measures of health and well-being than neighbors in the same age category, whereas no differences were found between younger gardeners and their younger neighbors. • Gardening had a significant positive effect on well-being, life satisfaction and loneliness.	Positive
Hawkins et al., 2011 [50]	Community gardeners: 25 Home gardeners: 21 Walkers: 25 Indoor exercisers: 23	Community gardeners: F = 32.0% Home Gardeners: 90.5% Walkers: 68.0% Indoor Exercisers: 87.0%	Community: Gardeners: 65.7 ± 9.1 Home Gardeners: 69.5 ± 7.7 Walkers: 62.4 ± 6.8 Indoor Exercisers: 72.9 ± 6.9	• BMI (Anthropometric assessment)	• Perceived stress • Perceived social support • Health-related quality of life	• Perceived Stress Scale (10-item) • Social Provisions Scale • International Physical Activity Questionnaire (IPAQ; short-form) • SF-36 v2 • Self-report of diagnosed illness • Self-report of current medication • Townsend Index Score	• Community gardeners reported significantly less perceived stress than participants of indoor exercise classes, which might be due to their engagement with nature and psychological restoration. • No significant differences between groups were found for self-reported levels of social support and physical activity.	Positive
Gerber et al., 2017 [49]	G:22 NG: 28	G:48.4% NG:51.6%	G:46 ± 14.32 NG:43.32 ± 15.69	NS	• Anxiety • Depression • Posttraumatic stress disorder • Somatization • Perceived social support	• Refugee Health Screener-15 • Patient Health Questionnaire-15 • Medical Outcomes Study Social Support Survey-19	• Gardeners and non-gardeners did not differ in levels of self-reported distress, symptoms of depression, anxiety and somatic complaints. • Gardeners reported greater social support than non-gardeners. • Age was positively associated with distress and somatization, whereas it was negatively associated with perceived support.	Positive only for social support.
Soga et al., 2017b [19]	G: 165 NG: 167	G: 31.9% NG: 58.2%	G: 61.9 ± 17.1 NG: 61 ± 16.6	• Perceived general health, subjective health, and BMI (self-reported height and weight)	• Social cohesion • Socio-demographic and lifestyle variables • Motivation, frequency and duration of gardening	• Perceived General Health (Single item question) • Subjective health complaints were measured with a 10-item question • Mental health was assessed using the 12-item General Health Questionnaire • Social Cohesion and Trust Scale • Socio-demographic and lifestyle items • Nature Relatedness Scale • The questionnaire for gardeners included a section about their motivation, frequency and duration of allotment gardening.	• Frequency and duration of gardening activities did not significantly influence self-reported health outcomes. • Community gardeners reported better general health, less somatic complaints, better mental health and greater social cohesion.	Positive
Booth et al., 2018 [53]	Regular gardeners: 16 Occasional gardeners: 43 NG: 56	Total: 47.8%	Total: 42.1%	• Physical health behaviours	• Mental health behaviours • Perceptions of the community • Levels of participation on community garden	• Self-rated health and Health behavior (Single item question) • Individual empowerment (two-item scale) • Well-being (five-item scale) • Psychological distress (six-item scale) • Life satisfaction (10-item scale) • Organizational and community empowerment was measured by asking respondents about their perception of neighborhood disorganization, their sense of community, and their perceived control at the organizational and community level • Ross et al.’s (2001) nine-item scale • Sense of community was measured using a 13-item scales (Peterson, Speer, & McMillan, 2008) • Community empowerment was measured using Schulz et al. (1995) four-item scale–Organizational empowerment was measured using Schulz et al. (1995) five item scale	• Regular and occasional participants reported better mental health. • Occasional Participants reported more vegetable intake, whereas regular participants reported more sense of community. • Participation in vegetable gardens was associated with increased levels of well-being and lower levels of distress. • The regularity of participation in horticultural activities did not affect well-being, which might indicate a selection bias (individuals with higher well-being are more likely to engage in community activities).	Positive
Mourão et al., 2019 [51]	G: 65	G: 43.1%	46–65: 47.7% 25–45 years: 36.9% >65 years: 36.9%	NS	• Life satisfaction • Subjective happiness • Subjective wellbeing	• Personal Well-Being Index—Adult (Bem-Estar Pessoal scale) • Subjective Happiness Scale	• Gardeners who visited the garden more frequently considered themselves more happier • Most relevant benefits of community gardening: occupation of free time, relaxation, and healthy food production. • Additional benefits of this activity: increased environmental awareness, change in diet habits, increased physical activity, socialization and interaction with others.	Positive
Koay & Dillon,2020 [52]	Individual/Home Gardening: 38 Community Gardening: 45 Non-Gardening Control: 28	Individual/Home Gardening: 84,2% Community Gardening: 44,4% Non-Gardening Control: 57,1%	Individual/Home Gardening: 43.76 ± 12.99 Community Gardening: 60.20 ± 13.27 Non-Gardening Control: 55.54 ± 11.62	NS	Connection to nature, resilience, perceived stress, subjective well-being, self-esteem, optimism and openness.	• Nature in Self Scale • Brief Resilience Scale • Perceived Stress Scale • Personal Wellbeing Index • Adult, Rosenberg Self-Esteem Scale • Life-Orientation Test-Revisited • Openness-to-Experience (10-item scale)	• After controlling for age and connection with nature, community gardeners reported significantly higher levels of subjective well-being and optimism than the control group and individual / domestic gardeners; • Resilience levels were significantly higher for the two groups of gardeners; no difference between groups was found for perceived stress, self-esteem and openness; • The connection with nature was positively correlated with resilience; resilience was positively correlated with levels of subjective well-being and negatively correlated with levels of perceived stress.	Positive

Notes: G, gardeners; NG, non-gardeners; F, Female; M, Male; SD, Standard deviation; BMI, Body mass index; NS, Not specified.

No study targeted only men or women, though gender representation within groups (gardeners *versus* non-gardeners) was highly variable among studies (Table 2). Only two studies indicated the range of participants’ age: 50+ years old [50] and between 18 and 100 years old [52]. The remaining studies provided the average age of the participants, usually above 40 years old for both gardeners and non-gardeners (Table 2).

### Community gardens and mental and physical well-being

Studies included in this literature review addressed two types of outcomes: physical and mental health and well-being. These were measured by asking participants to fill in specific questionnaires (Table 2). All studies assessed mental health and well-being, whereas physical health and well-being was covered in five out of the eight studies. Regarding physical health and well-being, respondents were generally asked to rate their general health status [19, 40, 49–51, 53, 54]. In one study, they were also asked about chronic conditions [40]. No study investigated musculoskeletal or osteoarticular injuries related to community gardening. Concerning mental health and well-being, gardeners and non-gardeners where asked about life satisfaction [40, 51, 53, 54], perceived stress [40, 50, 52], anxiety symptoms [49], depression symptoms [49], perceived social support [49, 50], health-related quality of life [50], and social contacts [19, 40]. The study conducted in Singapore also assessed connection with nature, resilience, subjective well-being, self-esteem, optimism and openness [52]. One study targeted Bhutanese refugees living in the USA and asked participants about posttraumatic stress and adjustment to the new country [49].

**Fig 2:**
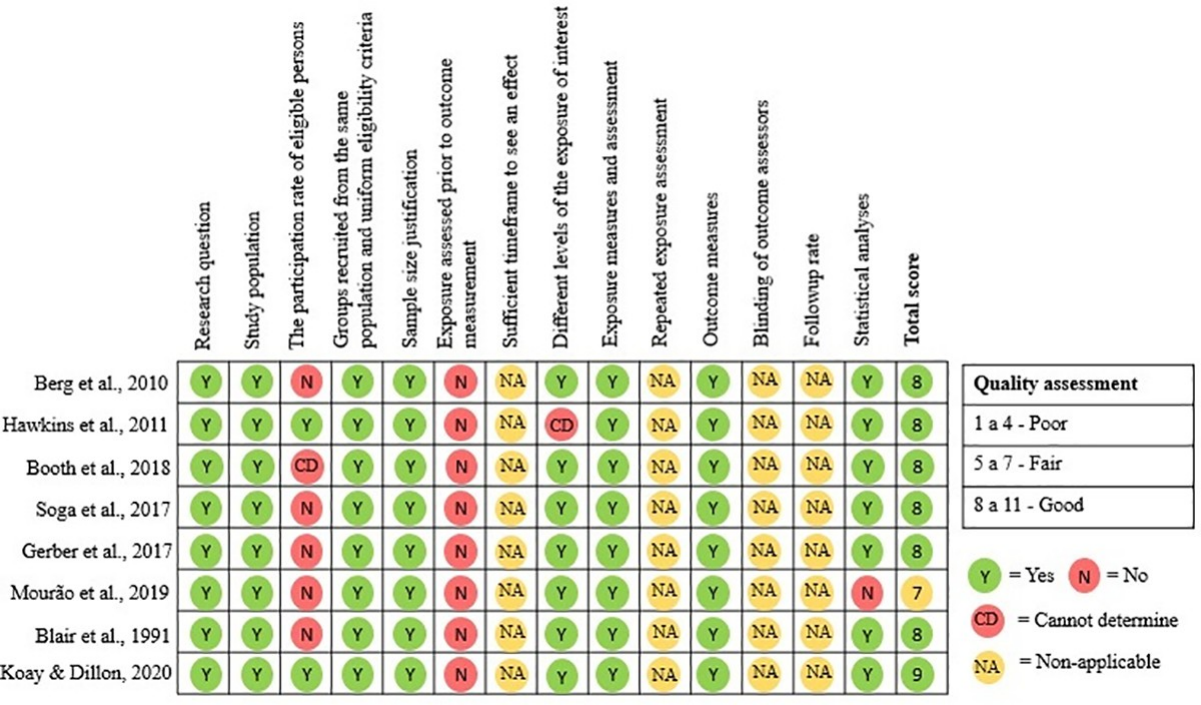
Quality assessment of the studies included in this literature review.

According to our quality assessment criteria, seven studies included in this literature review were rated as “good” and only one scored “fair” (Fig 2). Regarding the article scored as "fair", its results pointed out to a positive association between community gardens and physical and mental well-being [51]. Overall, a positive association between engaging in community gardening and physical and mental health and well-being was found in all studies included in this literature. A study addressing the mental health outcomes of community gardening among Nepali Bhutanese refugees living in the United States found perceived social support to be higher among gardeners than non-gardeners. However, no significant effect of community gardening on symptoms of depression, anxiety, somatic complains and adjustment to life in a new country was detected among these participants [49].

## Discussion

### Main findings

In this study, quantitative evidence on physical and mental health outcomes arising from engaging in community gardening was reviewed. Despite only eight studies met our inclusion criteria, their conclusions support the association between community gardening and positive physical (general health) and mental health (life satisfaction, happiness, mental health and social cohesion) outcomes among non-institutionalized individuals. No data about physical injuries (i.e., osteoarticular and/or musculoskeletal injuries) associated with engaging in community gardening activities were retrieved in the literature search.

### Positive health outcomes associated to community gardening activity

Overall, results here in provide evidence on the association between community gardening and positive health outcomes, irrespectively of participants’ gender, age, ethnicity, and country of residence. With regard to physical health, gardeners perceived their general health status to be better than community dwellers not involved in gardening activities [19, 40]. This might be due to the influence of gardening in health behaviors, namely regular physical activity [55], which is associated to a risk reduction for chronic conditions, such as cardiovascular disease, hypertension, cancer, obesity, but also to a reduction in the risk of premature death [56]. Indeed, gardening is considered to be a moderate intensity activity [41, 57], involving low to moderate intensity tasks [58] that proved sufficient for older adults to meet the recommendations on 30 minutes moderate intensity physical activity sessions, five (or more) days a week, if regularly undertaken [59]. Interestingly, one study included in this literature review reported differences in health outcomes between gardeners and non-gardeners only for those aged 62+ years—gardeners scored significantly better than non-gardeners, whereas no statistically significant differences were detected between younger gardeners and non-gardeners [40]. In a world getting older and characterized by an inverted age pyramid [60], community gardening seems a promising avenue to tackle age-related disability and promote healthy aging [61].

Apart from likely influencing health behaviors through increased physical activity, community gardening potentially impacts diet via increased consumption of fruit and vegetables [29, 62, 63]. Four studies included in this literature review provided data on the frequency of fruit and/or vegetable intake, which was higher for gardeners compared to non-gardeners [19, 40, 53, 54]. Moreover, growing vegetables for own consumption rated second concerning the motivations of Japanese community dwellers to engage in community gardening [19]. By successfully improving nutrition, community gardens not only contribute to reduce the risk of chronic diseases, such as cardiovascular disease and some cancers [64], but are also highly relevant to reduce inequalities in urban food systems [65]. As such, there has been growing interest in the role of these green spaces to increase access to nutritious food in the so-called ‘food deserts’, i.e., areas with limited access to affordable and nutritious food [29, 66, 67]. Evidence available from Rockford, Illinois, shows that community gardens also encompass diet benefits for non-gardeners because these individuals also have increased access to fruit and vegetables via shared production surplus from individual plots [66, 68]. Moreover, production from the cultivation of communal plots by volunteers engaged in local neighbourhood networks is also donated to social service organisations and deprived families, thus contributing to increase their access to nutritious food and reduce food inequalities [66].

All studies included in this literature review support a positive association between community gardening and mental health and well-being among non-institutionalized individuals. Overall, gardeners reported higher levels of life satisfaction [40, 51, 54], less perceived stress [40, 50], increased perceived social support [49] and social contacts [19, 40] than non-gardeners. Interestingly, perceived stress and social contacts were moderated by age among Dutch gardeners: community dwellers aged 62+ years engaged in gardening activities reported significantly lower stress levels and increased social contacts than non-gardeners (same age range), whereas no differences were found between younger gardeners and non-gardeners (62+ years) [40]. This finding is highly relevant under the context of healthy aging. As people age, their social network becomes narrower due to the combined effects of their reliance on stable and close relationships plus a decline in the establishment of new relationships [69]. As such, increased social contact by active participation in activities within the local neighbourhood, such as community gardening, has the potential to reduce loneliness feelings and increase mental health and well-being of older adults, although not restricted to this age group [30, 61, 70]. Community gardens provide a place for individuals to interact with other gardeners, neighbours, friends and family, thus contributing for broadening and strengthening of individual social networks, sometimes promoting intergenerational contacts [71] and social cohesion [72]. This encompasses positive impacts for mental health and well-being [73], in particular for vulnerable populations, such as older people [74] as previously considered. One study included in this literature review addressed the experiences of Bhutanese refugees during resettlement in the United States, by investigating and comparing several indicators of mental health and well-being between gardeners and non-gardeners [49]. Despite the two groups did not differ in levels of self-reported distress, symptoms of depression, anxiety and somatic complaints, gardeners reported significantly greater social support than non-gardeners [49]. Increased social support has been previously reported by refugees engaged in community gardening [75, 76], although only a few studies have been conducted up to now [74]. By gathering to grow vegetables and fruits, refugees interact with individuals with the same cultural background, which allows them to maintain ties to their culture of origin, but they are also provided with the opportunity for a smoothly inclusion process in the country of arrival by interacting with natives who also gather to gardening [75–77]. Interestingly, no differences for self-reported social support between community gardeners and home gardeners were found in one study included in this literature review (50). Further understanding on the association between engaging in community gardening *versus* home gardening and self-perceived social support will benefit from future comparative studies of these two activities.

Findings from this literature review are especially relevant given the current COVID-19 pandemic situation. The rapid spread of the SARS-CoV-2 virus brought a sudden change in the routine of the world population, and the year 2020 was characterized by lockdowns in several countries, as well as social containment and restrictions to mobility. Such abrupt disruptions in everyday life might negatively impact physical and mental health and well-being [78]. During periods of social isolation, easily accessible natural environments, such as community gardens, provide an adequate environment for individuals to engage in physical activity while relaxing [79, 80]. Outdoor green spaces in the neighbourhood where individuals can go, in a safer manner and complying with the recommendations from the health authorities, for time slots of 30–40 min everyday have an enormous potential to help build resilience and maintain physical and mental health and well-being [78]. Moreover, their role in complementing food shortages during crisis, such as during the World War II, is well known [81]. As such, community gardens potentially play a role in improving food security during the COVID-19 pandemic, which undoubtedly affected food systems [82].

### Community gardening-related physical injuries

No study assessing and/or reporting community gardening-related physical injuries, namely musculoskeletal and osteoarticular injuries, was retrieved in our literature search. This finding is quite striking given the large body of evidence available in the literature concerning physical injuries associated to agricultural practices and farming (e.g., [83–86]). For example, a systematic literature review addressing the prevalence of musculoskeletal disorders among farmers found that low back pain was the most frequently reported musculoskeletal disorder [87]. Injuries caused by hand tools manipulation, such as finger cuts, have also been frequently reported among farmers [86, 88]. Except for machinery, the types of hand tools used in farming and community gardening are potentially the same, e.g., shovel and sickle, which suggests that community gardeners might be exposed to the same types of injuries that farmers are. More research in this area is needed to disentangle between the physical health benefits *versus* potential risks of community gardening.

### Community gardens: A sustainable health promotion strategy

Human development and urbanization have generated a series of environmental problems, such as overconsumption of natural resources, water and air pollution, waste production [89, 90], and reduction of green spaces [89, 91]. These encompass major challenges and threats to human health and environmental sustainability [92–94]. Community gardening has the potential to contribute to achieve gains in human health and environmental sustainability, as pointed out in a growing body of literature (e.g., [95, 96] and also supported by results herein. By creating urban spaces where community dwellers gather to grow fruits and vegetables, public authorities are empowering the local communities and providing them with safer, enjoyable, all-inclusive settings that ease healthier choices, while fostering active participation in health and promoting the contact with nature in a sustainable manner, as envisaged in the Ottawa Charter for Health Promotion [97].

At the European level, one of the various actions under the European Green Deal, an action plan by the European Commission aimed at making the EU’s economy sustainable, is to ensure more sustainable food systems [98].To accomplish this, the creation of supportive food environments making easier to choose healthy and sustainable diets is central to achieve human health gains, thus reducing the economic burden of disease and the environmental impacts from food production [99]. This “Farm to Fork Strategy” establishes key goals to improve healthy lifestyles, health, and the environment by building a food chain that benefits both the consumer and the environment. Indeed, the recommendations under this H2020 Green Deal initiative aims at stimulating sustainable food production and processing practices; reducing the distance of the power chain between the source and the consumer; and increasing organic food production and food safety [99]. Under this context, community gardens potentially add valuable contributes to a more sustainable Europe concerning food system with focus in production and consumption.

Community gardens are an affordable and efficient, yet challenging, way to bring nature back to cities and potentially contribute to the provision of ecosystem services [100]. As green spaces, community gardens serve as a habitat for fauna and flora [101], being considered a potential reservoir of urban biodiversity [31, 102]. They also contribute to increase the proportion of permeable soil surface [103], filtering and storing water from the rain, thus contributing for floods’ prevention (Quayle, 2008). In addition, community gardens promote environmental education in urban areas [31, 100], offering a hands-on experience on ecological processes [104]. Thus, it is not surprising that interest in these green spaces has boomed in recent years, which often leaves community dwellers in waiting lists for a couple of years before being provided with a patch for them to cultivate [105]. Therefore, a great challenge in urban planning is now to increase the availability of these spaces. However, this cannot be done without considering the motivations that lead community dwellers to engage in community gardening [106], as well as to design and equip these green spaces with the infrastructures and tools that are needed for users to successfully profit from it [106].

### Strengths and limitations

This manuscript reviews quantitative evidence from cross-sectional studies on the association between community gardens and physical and mental health and well-being of the non-institutionalized population. However, given the cross-sectional study designs no causality relations can be ascertained.

To our knowledge, musculoskeletal and osteoarticular injuries have not been previously addressed in literature reviews. Despite no data was obtained on community gardening-related injuries, this is a relevant finding and indicates that more research in this realm is needed. However, since only a few articles were retrieved and are not representative of community gardening from any specific geographic region, any conclusions and generalizations should be taken cautiously. The few articles retrieved might be due to the language filter used—only studies published in English were considered. Nevertheless, considering that the great majority of the scientific peer-reviewed journals are published in English, we are confident that this methodological option did not significantly affect our results.

## Supporting information

S1 Checklist PRISMA 2009 checklist. (DOC)

S1 File Strategy to search for studies that investigate the evidence that community gardening contributes to increased physical and mental health and well-being of non-institutionalized persons. Details are provided only for one (PubMed) of the three databases surveyed (Web of Science and SCOPUS). The same search strategy was used for the remaining databases, and to meet the search requirements of each database required, some modifications were necessary in relation to the field tags.
(DOCX)
