# Supplementary material for: Evidence on the contribution of community gardens to promote physical and mental health and well-being of non-institutionalized individuals: A systematic review
Source: PLoS One. 2021 Aug 6;16(8):e0255621. doi: 10.1371/journal.pone.0255621 (PMC8345884; doi:10.1371/journal.pone.0255621)
Supplement: S1 File — Details are provided only for one (PubMed) of the three databases surveyed (Web of Science and SCOPUS). The same search strategy was used for the remaining databases, and to meet the search requirements of each database required, some modifications were necessary in relation to the field tags. (DOCX) [file pone.0255621.s002.docx]

**S1 File**. Strategy to search for studies that investigate the evidence that community gardening contributes to increased physical and mental health and well-being of non-institutionalized persons. Details are provided only for one (PubMed) of the three databases surveyed (Web of Science and SCOPUS). The same search strategy was used for the remaining databases, and to meet the search requirements of each database required, some modifications were necessary in relation to the field tags.

| **Database: PubMed**  The search was conducted on July 2-4, 2019 and updated on November 17-19, 2020 by using a pairwise combination of two blocks of both free-text and medical subject headings (MeSH) terms. | |
| --- | --- |
| **#** | **Searches** |
| 1 | Search (“Community garden*”[tiab] AND “Mental health”[tiab]) |
| 2 | Search (“Community garden*”[tiab] AND “Quality of life”[tiab]) |
| 3 | Search (“Community garden*”[tiab] AND *happiness[tiab]) |
| 4 | Search (“Community garden*”[tiab] AND “well*being”[tiab]) |
| 5 | Search (“Community garden*”[tiab] AND “Life satisfaction”[tiab]) |
| 6 | Search (“Community garden*”[tiab] AND “Satisfaction with life”[tiab]) |
| 7 | Search (“Community garden*”[tiab] AND “Psychological well*being”[tiab]) |
| 8 | Search (“Community garden*”[tiab] AND “Subjective well*being”[tiab]) |
| 9 | Search (“Community garden*”[tiab] AND “Musculoskeletal injur*”[tiab]) |
| 10 | Search (“Community garden*”[tiab] AND “Musculoskeletal condition*”[tiab]) |
| 11  12  13  14  15  16 | Search (“Community garden*”[tiab] AND “Osteo*articular injur*”[tiab])  Search (“Community garden*”[tiab] AND “Osteo*articular disease*”[tiab])  Search (“Community garden*”[tiab] AND Depression[tiab])  Search (“Community garden*”[tiab] AND Anxiety[tiab])  Search (“Community garden*”[tiab] AND Dysthymia[tiab])  Search (“Community garden*”[tiab] AND Loneliness[tiab]) |
| 17 | Search #1 OR #2 OR #3 OR #4 OR #5 OR #6 OR #7 OR #8 OR #9 OR #10  OR #11 OR #12 OR #13 OR #14 OR #15 OR #16 |
| 18 | English[la] |
| 19 | Search #17 AND #18 |
|  |  |
|  |  |
| 20 | Search (“Urban garden*”[tiab] AND “Mental health”[tiab]) |
| 21 | Search (“Urban garden*”[tiab] AND “Quality of life”[tiab]) |
| 22 | Search (“Urban garden*”[tiab] AND *happiness[tiab]) |
| 23 | Search (“Urban garden*”[tiab] AND “well*being”[tiab]) |
| 24 | Search (“Urban garden*”[tiab] AND “Life satisfaction”[tiab]) |
| 25 | Search (“Urban garden*”[tiab] AND “Satisfaction with life”[tiab]) |
| 26 | Search (“Urban garden*”[tiab] AND “Psychological well*being”[tiab]) |
| 27 | Search (“Urban garden*”[tiab] AND “Subjective well*being”[tiab]) |
| 28 | Search (“Urban garden*”[tiab] AND “Musculoskeletal injur*”[tiab]) |
| 29 | Search (“Urban garden*”[tiab] AND “Musculoskeletal condition*”[tiab]) |
| 30  31  32  33  34  35 | Search (“Urban garden*”[tiab] AND “Osteo*articular injur*”[tiab])  Search (“Urban garden*”[tiab] AND “Osteo*articular disease*”[tiab])  Search (“Urban garden*”[tiab] AND Depression[tiab])  Search (“Urban garden*”[tiab] AND Anxiety[tiab])  Search (“Urban garden*”[tiab] AND Dysthymia[tiab])  Search (“Urban garden*”[tiab] AND Loneliness[tiab]) |
| 36 | Search #20 OR #21 OR #22 OR #23 OR #24 OR #25 OR #26 OR #27 OR #28 OR #29 OR #30 OR #31 OR #32 OR #33 OR #34 OR #35 |
| 37 | English[la] |
| 38 | Search #36 AND #37 |
| 39 | Search (“allotment garden*”[tiab] AND “Mental health”[tiab]) |
| 40 | Search (“allotment garden*”[tiab] AND “Quality of life”[tiab]) |
| 41 | Search (“allotment garden*”[tiab] AND *happiness[tiab]) |
| 42 | Search (“allotment garden*”[tiab] AND “well*being”[tiab]) |
| 43 | Search (“allotment garden*”[tiab] AND “Life satisfaction”[tiab]) |
| 44 | Search (“allotment garden*”[tiab] AND “Satisfaction with life”[tiab]) |
| 45 | Search (“allotment garden*”[tiab] AND “Psychological well*being”[tiab]) |
| 46 | Search (“allotment garden*”[tiab] AND “Subjective well*being”[tiab]) |
| 47 | Search (“allotment garden*”[tiab] AND “Musculoskeletal injur*”[tiab]) |
| 48 | Search (“allotment garden*”[tiab] AND “Musculoskeletal condition*”[tiab]) |
| 49  50  51  52  53  54 | Search (“allotment garden*”[tiab] AND “Osteo*articular injur*”[tiab])  Search (“allotment garden*”[tiab] AND “Osteo*articular disease*”[tiab])  Search (“allotment garden*”[tiab] AND Depression[tiab])  Search (“allotment garden*”[tiab] AND Anxiety[tiab])  Search (“allotment garden*”[tiab] AND Dysthymia[tiab])  Search (“allotment garden*”[tiab] AND Loneliness[tiab]) |
| 55 | Search #39 OR #40 OR #41 OR #42 OR #43 OR #44 OR #45 OR #46 OR #47 OR #48 OR #49 OR #50 OR #51 OR #52 OR #53 OR #54 |
| 56 | English[la] |
| 57 | Search #55 AND #56 |
| 58 | Search (allotment[tiab] AND “Mental health”[tiab]) |
| 59 | Search (allotment[tiab] AND “Quality of life”[tiab]) |
| 60 | Search (allotment[tiab] AND *happiness[tiab]) |
| 61 | Search (allotment[tiab] AND “well*being”[tiab]) |
| 62 | Search (allotment[tiab] AND “Life satisfaction”[tiab]) |
| 63 | Search (allotment[tiab] AND “Satisfaction with life”[tiab]) |
| 64 | Search (allotment[tiab] AND “Psychological well*being”[tiab]) |
| 65 | Search (allotment[tiab] AND “Subjective well*being”[tiab]) |
| 66 | Search (allotment[tiab] AND “Musculoskeletal injur*”[tiab]) |
| 67 | Search (allotment[tiab] AND “Musculoskeletal condition*”[tiab]) |
| 68  69  70  71  72  73 | Search (allotment[tiab] AND “Osteo*articular injur*”[tiab])  Search (allotment[tiab] AND “Osteo*articular disease*”[tiab])  Search (allotment[tiab] AND Depression[tiab])  Search (allotment[tiab] AND Anxiety[tiab])  Search (allotment[tiab] AND Dysthymia[tiab])  Search (allotment[tiab] AND Loneliness[tiab]) |
| 74 | Search #58 OR #59 OR #60 OR #61 OR #62 OR #63 OR #63 OR #65 OR #66 OR #67 OR #68 OR #69 OR #70 OR #71 OR #72 OR #73 |
| 75 | English[la] |
| 76 | Search #74 AND #75 |
| 77 | Search (“community agriculture”[tiab] AND “Mental health”[tiab]) |
| 78 | Search (“community agriculture”[tiab] AND “Quality of life”[tiab]) |
| 79 | Search (“community agriculture”[tiab] AND *happiness[tiab]) |
| 80 | Search (“community agriculture”[tiab] AND “well*being”[tiab]) |
| 81 | Search (“community agriculture”[tiab] AND “Life satisfaction”[tiab]) |
| 82 | Search (“community agriculture”[tiab] AND “Satisfaction with life”[tiab]) |
| 83 | Search (“community agriculture”[tiab] AND “Psychological well*being”[tiab]) |
| 84 | Search (“community agriculture”[tiab] AND “Subjective well*being”[tiab]) |
| 85 | Search (“community agriculture”[tiab] AND “Musculoskeletal injur*”[tiab]) |
| 86 | Search (“community agriculture”[tiab] AND “Musculoskeletal condition*”[tiab]) |
| 87  88  89  90  91  92 | Search (“community agriculture”[tiab] AND “Osteo*articular injur*”[tiab])  Search (“community agriculture”[tiab] AND “Osteo*articular disease*”[tiab])  Search (“community agriculture”[tiab] AND Depression[tiab])  Search (“community agriculture”[tiab] AND Anxiety[tiab])  Search (“community agriculture”[tiab] AND Dysthymia[tiab])  Search (“community agriculture”[tiab] AND Loneliness[tiab]) |
| 93 | Search #77 OR #78 OR #79 OR #80 OR #81 OR #82 OR #83 OR #84 OR #85 OR #86 OR #87 OR #88 OR #89 OR #90 OR #91 OR #92 |
| 94 | English[la] |
| 95 | Search #93 AND #94 |
| 96 | Search (“agricultural allotment”[tiab] AND “Mental health”[tiab]) |
| 97 | Search (“agricultural allotment”[tiab] AND “Quality of life”[tiab]) |
| 98 | Search (“agricultural allotment”[tiab] AND *happiness[tiab]) |
| 99 | Search (“agricultural allotment”[tiab] AND “well*being”[tiab]) |
| 100 | Search (“agricultural allotment”[tiab] AND “Life satisfaction”[tiab]) |
| 101 | Search (“agricultural allotment”[tiab] AND “Satisfaction with life”[tiab]) |
| 102 | Search (“agricultural allotment”[tiab] AND “Psychological well*being”[tiab]) |
| 103 | Search (“agricultural allotment”[tiab] AND “Subjective well*being”[tiab]) |
| 104 | Search (“agricultural allotment”[tiab] AND “Musculoskeletal injur*”[tiab]) |
| 105 | Search (“agricultural allotment”[tiab] AND “Musculoskeletal condition*”[tiab]) |
| 106  107  108  109  110  111 | Search (“agricultural allotment”[tiab] AND “Osteo*articular injur*”[tiab])  Search (“agricultural allotment”[tiab] AND “Osteo*articular disease*”[tiab])  Search (“agricultural allotment”[tiab] AND Depression[tiab])  Search (“agricultural allotment”[tiab] AND Anxiety[tiab])  Search (“agricultural allotment”[tiab] AND Dysthymia[tiab])  Search (“agricultural allotment”[tiab] AND Loneliness[tiab]) |
| 112 | Search #96 OR #97 OR #98 OR #99 OR #100 OR #101 OR #102 OR #103 OR #104 OR #105 OR #106 OR #107 OR #108 OR #109 OR #110 OR #111 |
| 113 | English[la] |
| 114 | Search #112 AND #113 |
| 115 | Search (“household garden*”[tiab] AND “Mental health”[tiab]) |
| 116 | Search (“household garden*”[tiab] AND “Quality of life”[tiab]) |
| 117 | Search (“household garden*”[tiab] AND *happiness[tiab]) |
| 118 | Search (“household garden*”[tiab] AND “well*being”[tiab]) |
| 119 | Search (“household garden*”[tiab] AND “Life satisfaction”[tiab]) |
| 120 | Search (“household garden*”[tiab] AND “Satisfaction with life”[tiab]) |
| 121 | Search (“household garden*”[tiab] AND “Psychological well*being”[tiab]) |
| 122 | Search (“household garden*”[tiab] AND “Subjective well*being”[tiab]) |
| 123 | Search (“household garden*”[tiab] AND “Musculoskeletal injur*”[tiab]) |
| 124 | Search (“household garden*”[tiab] AND “Musculoskeletal condition*”[tiab]) |
| 125  126  127  128  129  130 | Search (“household garden*”[tiab] AND “Osteo*articular injur*”[tiab])  Search (“household garden*”[tiab] AND “Osteo*articular disease*”[tiab])  Search (“household garden*”[tiab] AND Depression[tiab])  Search (“household garden*”[tiab] AND Anxiety[tiab])  Search (“household garden*”[tiab] AND Dysthymia[tiab])  Search (“household garden*”[tiab] AND Loneliness[tiab]) |
| 131 | Search #115 OR #116 OR #117 OR #118 OR #119 OR #120 OR #121 OR #122 OR #123 OR #124 OR #125 OR #126 OR #127 OR #128 OR #129 OR #130 |
| 132 | English[la] |
| 133 | Search #131 AND #132 |
| 134 | Search (“roof*top garden*”[tiab] AND “Mental health”[tiab]) |
| 135 | Search (“roof*top garden*”[tiab] AND “Quality of life”[tiab]) |
| 136 | Search (“roof*top garden*”[tiab] AND *happiness[tiab]) |
| 137 | Search (“roof*top garden*”[tiab] AND “well*being”[tiab]) |
| 138 | Search (“roof*top garden*”[tiab] AND “Life satisfaction”[tiab]) |
| 139 | Search (“roof*top garden*”[tiab] AND “Satisfaction with life”[tiab]) |
| 140 | Search (“roof*top garden*”[tiab] AND “Psychological well*being”[tiab]) |
| 141 | Search (“roof*top garden*”[tiab] AND “Subjective well*being”[tiab]) |
| 142 | Search (“roof*top garden*”[tiab] AND “Musculoskeletal injur*”[tiab]) |
| 143 | Search (“roof*top garden*”[tiab] AND “Musculoskeletal condition*”[tiab]) |
| 144  145  146  147  148  149 | Search (“roof*top garden*”[tiab] AND “Osteo*articular injur*”[tiab])  Search (“roof*top garden*”[tiab] AND “Osteo*articular disease*”[tiab])  Search (“roof*top garden*”[tiab] AND Depression[tiab])  Search (“roof*top garden*”[tiab] AND Anxiety[tiab])  Search (“roof*top garden*”[tiab] AND Dysthymia[tiab])  Search (“roof*top garden*”[tiab] AND Loneliness[tiab]) |
| 150 | Search #134 OR #135 OR #136 OR #137 OR #138 OR #139 OR #140 OR #141 OR #142 OR #143 OR #144 OR #145 OR #146 OR #147 OR #148 OR #149 |
| 151 | English[la] |
| 152 | Search #150 AND #151 |
| 153 | Search (“roof*top agriculture”[tiab] AND “Mental health”[tiab]) |
| 154 | Search (“roof*top agriculture”[tiab] AND “Quality of life”[tiab]) |
| 155 | Search (“roof*top agriculture”[tiab] AND *happiness[tiab]) |
| 156 | Search (“roof*top agriculture”[tiab] AND “well*being”[tiab]) |
| 157 | Search (“roof*top agriculture”[tiab] AND “Life satisfaction”[tiab]) |
| 158 | Search (“roof*top agriculture”[tiab] AND “Satisfaction with life”[tiab]) |
| 159 | Search (“roof*top agriculture”[tiab] AND “Psychological well*being”[tiab]) |
| 160 | Search (“roof*top agriculture”[tiab] AND “Subjective well*being”[tiab]) |
| 161 | Search (“roof*top agriculture”[tiab] AND “Musculoskeletal injur*”[tiab]) |
| 162 | Search (“roof*top agriculture”[tiab] AND “Musculoskeletal condition*”[tiab]) |
| 163  164  165  166  167  168 | Search (“roof*top agriculture”[tiab] AND “Osteo*articular injur*”[tiab])  Search (“roof*top agriculture”[tiab] AND “Osteo*articular disease*”[tiab])  Search (“roof*top agriculture”[tiab] AND Depression[tiab])  Search (“roof*top agriculture”[tiab] AND Anxiety[tiab])  Search (“roof*top agriculture”[tiab] AND Dysthymia[tiab])  Search (“roof*top agriculture”[tiab] AND Loneliness[tiab]) |
| 169 | Search #153 OR #154 OR #155 OR #156 OR #157 OR #158 OR #159 OR #160 OR #161 OR #162 OR #163 OR #164 OR #165 OR #166 OR #167 OR #168 |
| 170 | English[la] |
| 171 | Search #169 AND #170 |
| 172 | Search (“roof*top farm*”[tiab] AND “Mental health”[tiab]) |
| 173 | Search (“roof*top farm*”[tiab] AND “Quality of life”[tiab]) |
| 174 | Search (“roof*top farm*”[tiab] AND *happiness[tiab]) |
| 175 | Search (“roof*top farm*”[tiab] AND “well*being”[tiab]) |
| 176 | Search (“roof*top farm*”[tiab] AND “Life satisfaction”[tiab]) |
| 177 | Search (“roof*top farm*”[tiab] AND “Satisfaction with life”[tiab]) |
| 178 | Search (“roof*top farm*”[tiab] AND “Psychological well*being”[tiab]) |
| 179 | Search (“roof*top farm*”[tiab] AND “Subjective well*being”[tiab]) |
| 180 | Search (“roof*top farm*”[tiab] AND “Musculoskeletal injur*”[tiab]) |
| 181 | Search (“roof*top farm*”[tiab] AND “Musculoskeletal condition*”[tiab]) |
| 182  183  184  185  186  187 | Search (“roof*top farm*”[tiab] AND “Osteo*articular injur*”[tiab])  Search (“roof*top farm*”[tiab] AND “Osteo*articular disease*”[tiab])  Search (“roof*top farm*”[tiab] AND Depression[tiab])  Search (“roof*top farm*”[tiab] AND Anxiety[tiab])  Search (“roof*top farm*”[tiab] AND Dysthymia[tiab])  Search (“roof*top farm*”[tiab] AND Loneliness[tiab]) |
| 188 | Search #172 OR #173 OR #174 OR #175 OR #176 OR #177 OR #179 OR #180 OR #181 OR #182 OR #183 OR #184 OR #185 OR #186 OR #187 OR #188 |
| 189 | English[la] |
| 190 | Search #188 AND #189 |
